# Use of rectal balloon spacer in patients with localized prostate cancer receiving external beam radiotherapy

**DOI:** 10.1016/j.tipsro.2024.100237

**Published:** 2024-01-18

**Authors:** Paulo Costa, Joana Vale, Graça Fonseca, Adelina Costa, Michael Kos

**Affiliations:** a CUF Porto Instituto, Rua Fonte das Sete Bicas, 170 - Piso -1 – 4460-188 SENHORA DA HORA, Porto Portugal; b Brachytherapy Radiation Specialists Summit Cancer, 6506 Regal Ct., Reno, NV 99223, USA

**Keywords:** Prostate cancer, Perirectal spacer, Radiation therapy, Organ at risk, Balloon spacer

## Abstract

**Objective:**

To evaluate the efficacy of the balloon spacer when used to reduce the radiation dose delivered to the rectum in prostate cancer patients undergoing external beam radiotherapy.

**Method:**

A single center retrospective analysis including 75 PC patients with localized T1-T3a disease who received balloon spacer followed by EBRT. Pre- and post-implantation computed tomography (CT) scans were utilized for treatment planning for standard EBRT (78–81 Gy in 1.8–2 Gy fractions). Rectal dosimetry was assessed using DVHs, and toxicities were graded with CTCAE v.4.

**Results:**

A median (IQR) prostate-rectum separation resulted in 1.6 cm (1.4–2.0) post balloon spacer implantation. Overall, 90.6 % (68/75) of patients had a clinically significant 25 % relative reduction in the rectal with a median relative reduction of 91.8 % (71.2–98.6 %) at rV70. Three (4.0 %) patients reported mild procedural adverse events, anal discomfort and dysuria. Within 90 days post-implantation, five patients (6.67 %) and 1 patient (1.33 %) reported grade 1 and grade 2 rectal toxicities (anal pain, constipation, diarrhea and hemorrhoids). Genitourinary (GU) grade 1 toxicity was reported in 37 patients (49.33 %), with only one patient (1.33 %) experiencing grade 2 GU toxicity. No grade ≥ 3 toxicity was reported.

**Conclusion:**

Balloon spacer implantation effectively increased prostate-rectum separation and associated with dosimetric gains EBRT for PC stage T1-T3a. Further controlled studies are required to ascertain whether this spacer allows for radiotherapy dose escalation and minimizes gastrointestinal (GI) toxicity.

## Highlights

• The use of the balloon rectal spacer improves rectal dosimetry resulted in a median relative rectal dose reduction of 91.8%.
• The median separation between the prostate and rectum achieved 1.6 cm post-implant, consistent with the reproducibility and pre-formed volume of the balloon.
• The balloon spacer was well-tolerated, with only mild procedural adverse events and low rates of acute and late gastrointestinal and genitourinary toxicities.
• The balloon spacer insertion technique keeps the needle away from the rectal wall. It includes the use of a blunt-dissection tip which reduces the risk of rectal injuries.
• The balloon spacer is well visualized on all imaging modalities, including TRUS, CT and MRI which facilitates accurate placement and monitoring of the spacer during radiation therapy.

## Introduction

Prostate cancer (PC) is a commonly diagnosed malignancy in men and the 5th commonest cause of cancer death globally [1]. Radiotherapy (RT) is an accepted primary treatment for men with localized or locally advanced PC [2] where reported outcomes are comparable with radical surgery in stratified risk groups [3]. External beam radiation therapy (EBRT) for prostate cancer has been developing rapidly with an increasingly safe radiation dose delivery. Nevertheless, the main challenge remains to deliver adequately high radiation dose to the prostate in order to achieve tumor control, while meeting the planning treatment constraints to ensure the safety of adjacent healthy tissues. Dose escalation is limited by toxicity to the surrounding healthy tissues with both acute and late genitourinary (GU) as well as gastrointestinal (GI) side-effects from this approach adversely affecting quality of life [4], [5].

There have been important modifications and alternatives in the delivery of prostate RT over recent decades with the introduction of conformal techniques, image-guided radiation therapy (IGRT) intensity-modulated radiation therapy (IMRT), volumetric modulated arc therapy (VMAT) and stereotactic body radiation therapy (SBRT), each with the principal aims of improving cancer-specific outcome through dose escalation whilst limiting surrounding toxicity.

Despite these advances, the rectum remains the critical organ at risk for damage with prostate cancer RT because of its close anatomical relationship to the posterior prostatic wall. In this regard, using dose escalated RT (up to 78 Gy) acute and chronic Grade 2 rectal toxicity has been variably reported in between 3 and 20 % and 5–21 % of treated cases, respectively [6], [7] with the risk of late rectal toxicity correlating with the volume of the rectal wall that receives a total dose > 70 Gy [8], [9]. The spacer approach which separates the prostate from the anterior rectal wall has been designed to reduce the rectal dose without compromising the prostate planning target volume (PTV), thereby potentially resulting in relatively low rates of rectal toxicity with higher RT dose delivery [10]. A variety of spacer materials have been used, each with different physical characteristics and dosimetric profiles including collagen implants [11] polyethylene glycol-based hydrogel (SpaceOAR, Boston Scientific) [12], and hyaluronic acid (HA, Barrigel, Palette Life Science) [13].

These spacers are made of biodegradable materials that absorbed by the patient’s body over time. Both HA and SpaceOAR are injected transperineally between the prostate and anterior rectum, then harden and cannot be repositioned. This approach using biomaterials inserted between the prostate and the rectum has been shown to consistently reduce the rectal dose in those treated with EBRT [14], [15].

The balloon spacer is a biodegradable balloon made of poly (L-Lactide-cocaprolactone) which is a co-polymer of Poly Lactide acid and epsilon Caprolactone (BioProtect Balloon Implant System, BioProtect Ltd) inflated with saline, providing around 18 mm space height [16], and can be deflated and repositioned if needed, both laterally and along the distal /proximal planes, for optimal uniformity of spacing.

This retrospective study reports a single institution experience of 75 patients with localized (T1-T3a) PC receiving EBRT who were managed with the balloon spacer. The primary objective of the study was to determine the dosimetric gain of the rectum associated with the balloon spacer. The secondary objectives were to evaluate the technical feasibility of the balloon‘s implantation and evaluation of rectal toxicities related to the RT and procedure according to the National Cancer Institute – Common Terminology Criteria for Adverse Events (CTCAE, version 4.0) [17].

## Patients and methods

### Patient inclusions, balloon implantation, dosimetry comparisons

The analysis was approved by the Ethics Committee of the institute (No. HCP/CES – 11/2).The study population included all patients managed between January 2015 and September 2020, diagnosed with prostate cancer that is locally confined or extracapsular with no posterior extension (i.e., not involving the rectum, with a clinical PC stage T1-T3a) who were implanted with the balloon spacer and with a planned treatment regime of radiotherapy by means of EBRT.

Baseline computed tomography (CT) scan was taken before implantation of the balloon spacer. The balloon placement procedure was previously described by Vanneste et al [18]. Briefly, the implantation was performed transperineally with patients in the dorsal lithotomy position. Anesthesia was either general (n = 52) or local (n = 23) using 2 % lidocaine that was injected into the perineal skin, to the prostate apex left and right obturator plexus. A foley catheter was used in all procedures. The insertion of fiducial markers into the prostate was followed by the balloon spacer implantation into the anterior perirectal space between Denonvilliers’ fascia and the anterior rectal wall. The implantation was done by radiation oncologists, and under transrectal ultrasound (TRUS) guidance using the transperineal approach.

The balloon was implanted by using a blunt tip dilator and an introducer sheath that was inserted via a small perineal incision creating a working channel along the plane from prostate apex to base. After proper positioning, the balloon was inflated with sterile physiological saline solution, and sealed.

Treatment planning was done using post implantation CT scan taken about one week after balloon implantation. All CT scans were done in the same position with similar patient’s preparations including empty bowel and bladder.

The prescribed dose delivery to the prostate was 80 Gy (40 fractions) in 59 patients, 60 Gy (20 fractions) in 7 patients, 84 Gy (42 fractions) in 2 patients, 82 Gy (41 fractions) in 2 patients, 78 Gy (39 fractions) in 2 patients, 70 Gy (35 fractions) in 2 patients and 74 Gy (37 fractions) in one patient. Pelvic lymph nodes irradiation was performed with a conventional 2 Gy/ fraction to a total dose of 46 Gy in 28 patients.

Patients with low, intermediate favorable-risk prostate cancer, medical contra-indication or individual refusal didn’t receive concomitant hormone therapy.

Clinical Target Volume (CTV) was defined according to the prostate stratification risk disease [19]. CTV as prostate only for low-risk patients, prostate and proximal seminal vesicles (1 cm) for the intermediate risk group, and prostate with seminal vesicles (2 cm) for the high-risk group. PTV was generated with a CTV to PTV margin expansion of 5 mm. For T3a patients with high-risk disease, PTV was generated with additional 3 mm expansion to the affected side with an anisotropic expansion planning tool. Daily Cone beam CT acquisition was performed prior to daily treatments.

Pelvic lymph nodes were treated if the risk of pathologically positive nodes according to the Roach formula would be greater than 20 % [20]. Rectum dose goal constraints were as V50 < 50 %, V60 < 35 %, V65 < 25 %, V70 < 20 % and V75 < 15 %. The contoured organs at risk (OARs) included the bladder, rectum, femoral heads and the penile bulb. The rectum was considered the principal OAR with its volume defined at CT-pre and CT-post scans (length from PTV + 1.0 cm cranial and caudal). The dosimetric effect of the balloon spacer was evaluated by comparing the median and the percentage differences in the rectal and bladder doses that received 30 Gy up to 80 Gy, representing 50.0 %, 62.5 %, 75.0 %, 87.5 % and 100 % of the prescribed dose, including analysis of both the pre- and post-planning dose volume histograms (DVH).

The reduction of the rectal dose was calculated such that D_pre_ is the rectum dose at baseline and D_post_ is the rectum dose following balloon spacer implantation [16].

For all RT plans, the near-minimal dose (D98%) exceeded 95 % with the near-maximal dose (D2%) < 107 % of the prescribed dose in accordance with the ICRU-83 recommendations [21].

### Quantifying the perirectal space

**Fig. 1:**
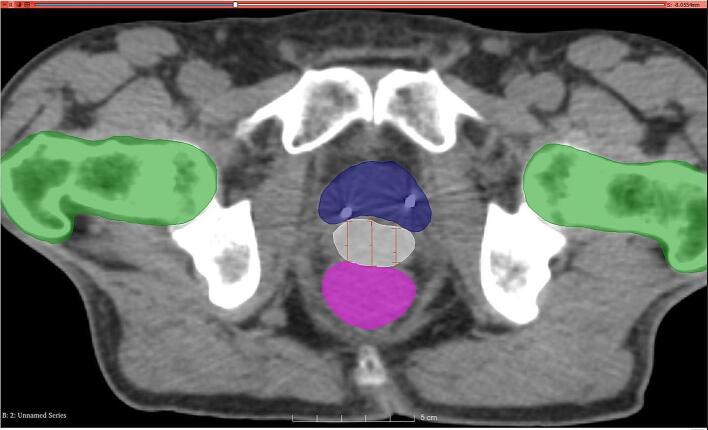
CT scan of balloon spacer between the rectum and prostate in axial view. Spacer measured at midline, to that 1 cm bilaterally from midline.

The CT-post for all treated patients was used to measure the distance between the median locations of the posterior prostatic capsule and the anterior rectal wall (Fig. 1). The prostate-rectum distance was measured at 3 discrete positions, namely: mid-gland level (center of CTV), 1 cm laterally left and right from the center of the CTV (corresponding to the position of the midline of the mid-gland).

### Safety data collection and follow-up

All subjects included in this study were evaluated for acute GI and GU toxicities from balloon implantation through 3 months after implantation.

### Statistical analysis

All statistical analyses were performed using SAS, version 9 (SAS Institute, Cary, NC). Numerical variables (patients demographics, volumes and radiation exposure) were tabulated using number of patients median and interquartile range (25th to 75th percentile) for continuous variables and proportions for categorical variables. Categorical variables were tabulated using the number and percentage of patients. Comparisons between pre- and post-implantation plan features and doses were performed using the Wilcoxon signed rank test which was a non-parametric statistical test that compares the two groups. Two-sided P values < 0.05 were considered statistically significant.

## Results

### Radiotherapy treatment plans and spacer impact

After approval by the ethical committee, 75 PC patients implanted with the balloon spacer were retrospectively included in this study with the last case completing treatment at the end of September 2020.

Additional clinicopathologic features of the cohort are shown in Table 1.

**Table 1:** Clinicopathologic features of the cohort (n = 75).

**Age (Years)**	
Median [IQR]	75.9 [70.7–78.9]
**PSA (ng/mL)**	
Median [IQR]	8.1 [5.1–10.5]
**Gleason Grade, n (%)**	
5 (2 + 3)	1 (1.3 %)
6 (3 + 3)	8 (10.7 %)
7 (3 + 4)	20 (26.7 %)
7 (4 + 3)	15 (20.0 %)
8 (3 + 5)	3 (4.0 %)
8 (4 + 4)	17 (22.7 %)
9 (4 + 5)	10 (13.3 %)
9 (5 + 4)	1 (1.3 %)
**Tumor Stage, n (%)**	
T1-T2	67 (89.3 %)
T3	8 (10.7 %)
**Irradiation Dose, n (%)**	
57 Gy	1 (1.3 %)
60 Gy	5 (6.6 %)
70 Gy	2 (2.6 %)
74 Gy	1 (1.3 %)
78 Gy	1 (1.3 %)
80 Gy	60 (80.0 %)
82 Gy	2 (2.6 %)
84 Gy	2 (2.6 %)

Abbreviations: PSA, prostate-specific antigen; RT, Radiation therapy; IQR, interquartile range.

**Fig. 2:**
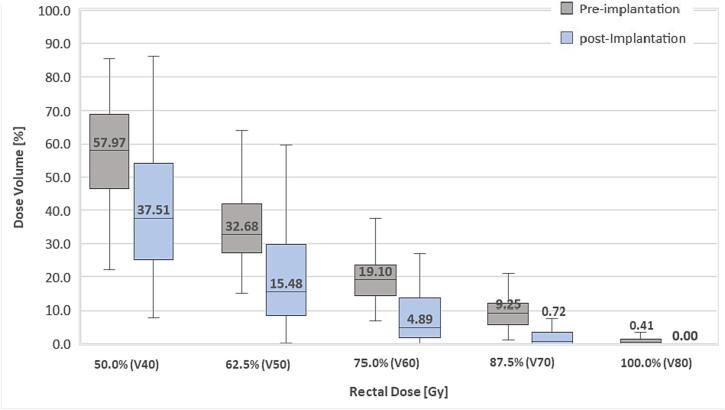
**Dose-volume Box-and-Whisker plot showing median rectal doses for 75 patients on pre- and post-implantation treatment plans.** Difference in sparing of the rectum with radiation treatment of V40, V50, V60, V70 and V80, representing 50.0%, 62.5%, 75.0%, 87.5% and 100.0% of the prescribed dose. The 25th and 75th percentiles are provided at the bottom and top of the boxes, respectively. The horizontal lines inside the boxes indicate median values.

**Fig. 3:**
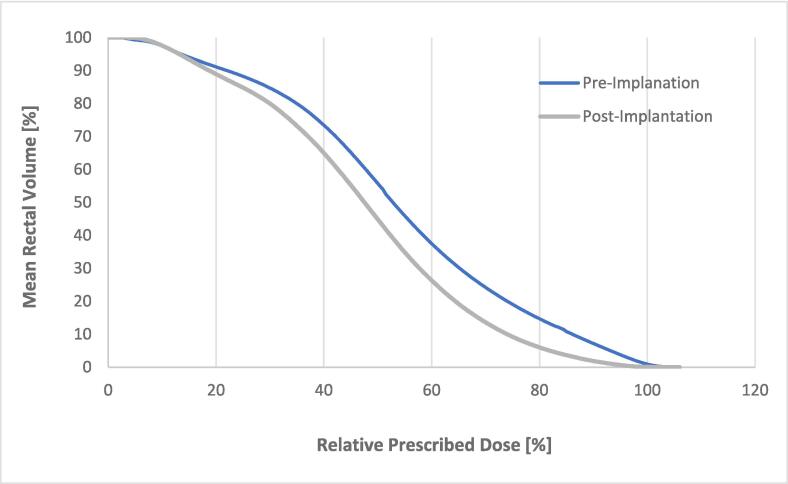
Mean Relative Rectal Dose-Volume Histograms before and following balloon spacer implantation; n = 75.

The median (IQR) dosimetric parameters of the PTV, rectum and bladder are shown in Table 2. All dose–volume constraints were satisfactorily met for all treatment plans. Rectal dose-volume measurements were significantly lower post implantation (both absolute and relative) compared to pre balloon implantation (p < 0.0001), also Fig. 2. The results showed that 68/75 patients (90.6 %) had a clinically significant 25 % relative reduction in the rectal V70, and 11.0 % achieved a 100 % rectal V70 reduction. Fig. 3 shows the mean DVH histogram curves for the rectum in the available cohort before and after spacer insertion. Although reductions were seen in the bladder dose, the data should be interpreted with caution because the volume of the bladder which received 80 Gy was not systematically controlled through the process.

**Table 2:** Comparison of pre- and post- implantation treatment plans. The rectum and bladder at V40, V50, V60, V70 and V80 = rectum or bladder volumes receiving 50.0 %, 62.5 %, 75.0 %, 87.5 % and 100.0 % of the prescribed dose; n = 75 patients.

	**Parameter**	**% Pre spacer** **(Median [IQR])**	**% Post Spacer** **(Median [ IQR])**	**Change from baseline** **(Median [ IQR])**	**Percentage reduction** **(Median [ IQR])**	p-value*
**PTV**	**Volume (cc)**	96.1 [78.8–116.6]	104.2 [81.2–120.1]	−6.8 [-13.3–1.5]	−2.9 [-10.7–1.4]	0.21
**D_mean_ (Gy)**		83.4 [81.7–101.9]	82.4 [80.8–102.4]	0.1 (-0.8–1.6]	0.2 [-1.0–1.9]	0.22
**Rectum**	**Volume (cc)**	51.1 [43.3–58.5]	54.8 [46.0–67.7]	−3.6 [-6.5–1.8]	−7.7 [-14.6–3.4]	0.15
	**50.0 % (V40)**	57.9 [46.5–68.8]	37.5 [25.1–54.0]	20.1 [5.4–28.2]	32.3 [9.3–50.9]	<0.0001
	**62.5 % (V50)**	32.6 [27.2–42.0]	15.4 [8.5–29.6]	17.2 [8.3–27.0]	53.7 [18.3–72.5]	<0.0001
	**75.0 % (V60)**	19.1 [14.2–1.9]	4.8 [1.9–13.5]	14.3 [7.4–18.1]	72.7 [37.3–87.3]	<0.0001
	**87.5 % (V70)**	9.2 [5.8––11.9]	0.7 [0.1–3.5]	8.4 [4.1–10.5]	91.8 [71.2–98.6]	<0.0001
	**100 % (V80)**	0.4 [0.0–1.4]	0.0 [0.0–0.0]	0.4 [0.0–1.4]	100.0 [100.0–100.0]	<0.0001
**Bladder**	**Volume (cc)**	96.5 [68.2–151.8]	78.2 [59.6–106.5]	21.1 [-11.5–75.6]	19.5 [-18.3–48.1]	0.01
	**50.0 % (V40)**	37.0 [24.0–57.1]	36.0 [24.0–57.1]	0.8 [-7.7–10.7]	1.0 [-29.2–23.8]	0.75
	**62.5 % (V50)**	21.6 [14.4–34.1]	22.4 [15.3–32.0]	0.6 [-6.9–8.7]	1.9 [-42.8–30.4]	0.45
	**75.0 % (V60)**	11.3 [7.6–17.8]	11.0 [7.7–16.8]	0.2 [-4.6–4.3]	2.7 [-58.5–41.2]	0.47
	**87.5 % (V70)**	5.2 ([2.6–6.9]	4.6 [2.2–7.3]	0.5 [-3.0–2.7]	11.5 [-60.8–48.7]	0.62
	**100 % (V80)**	0.1 [0.0–0.4]	0.0 [0.0–0.2]	0.1 [0.0–0.3]	89.7 [–23.8–100.0]	<0.001

Abbreviations: IQR, interquartile range; PTV, planning target volume.

* p-values are calculated using Wilcoxon matched-paired signed-rank test.

### Prostate-Rectum distance separation

**Fig. 4:**
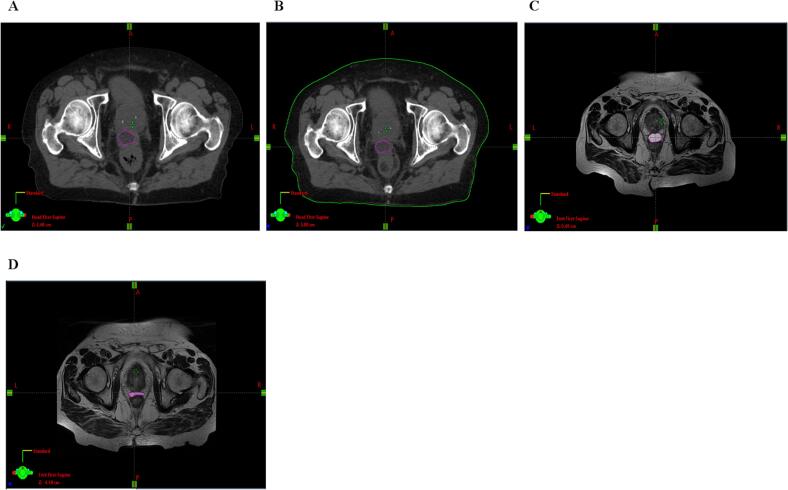
CT and MR images of post-balloon implantation (pink color) at different time points: A) CT simulation done at post balloon implantation, B) CT image at the end of radiotherapy, C) MR image at end of radiotherapy, D) MR image at 8 weeks from the end of radiation therapy. (For interpretation of the references to color in this figure legend, the reader is referred to the web version of this article.)

Perirectal distance was calculated from CT images taken after the balloon spacer placement. The median (IQR) perirectal distance after spacer placement was 1.76 (0.8–2.0) cm, 1.77 (0.8–2.1) cm, and 1.57 (0.8–1.8) cm at mid-gland (center of CTV), and 1 cm laterally left and right, respectively (Table 3). The median measurement of the 3 perirectal distances was 1.60 (1.4–2.0) cm. There were no observed substantial variations in the balloon volume from CT simulation, through daily cone beam CT to last fraction CT (Fig. 4).

**Table 3:** Measurement of the spacers and prostate-rectum interspace as measured on the planning CT (n = 75). Abbreviations: = IQR, interquartile range.

**Measurement Site**	**Prostate-Rectum Distance(cm)**
**1 cm laterally right** Median [IQR]	1.57 [0.80–1.80]
**Mid-gland** Median [IQR]	1.76 [0.80–2.02]
**1 cm laterally left** Median [IQR]	1.77 [0.80–2.10]

### Adverse events

The balloon insertion was successful in all patients and was well tolerated without any major complications, infections, or rectal bleeding. Three (4.0 %) patients reported mild (grade 1) procedural adverse events (anal discomfort, dysuria). Overall, within 3 months from implantation, five patients (6.67 %) and 1 patient (1.33 %) reported grade 1 and grade 2 rectal toxicities (anal pain, constipation, diarrhea, and hemorrhoids). GU grade 1 toxicity was reported in 37 patients (49.33 %) which included dysuria, hematuria, urinary pain and urinary frequency. Only one patient (1.33 %) reported grade 2 GU toxicity (urinary retention). No grade ≥ 3 toxicity was reported.

## Discussion

Interstitial spacers constitute a method designed to achieve rectal dose reduction during the delivery of radiotherapy for prostate cancer. In the current study, balloon spacer implantation resulted in a rectal dose reduction in each of the 75 patients with no technical difficulties and a very low incidence of mild (Grade 1) procedure-related adverse events.

In this study, we demonstrated a statistically significant rectal dose reduction for rV40 to rV80 relative to the pre-balloon implantation plans. The median absolute dose at rV70 was 6.7 % in the post implantation plans, representing a median dose reduction of 91.8 % (Table 2).

Our results appear to be comparable with other studies that have reported significant reduction in rectal radiation dose to the rectum using rectal spacers [12], [13]. A multicenter randomized controlled trial conducted in the United States[12], reported on high level of successful hydrogel spacer placement with a 73.3 % mean relative reduction in the rectal V70. The dosimetric gain in rectal V70 in our cohort is also in-line with more recent publications [13], [22], [23] reporting on mean rectal dose reduction of 85.0 % (±20.9 %), median rectal dose reduction of 91.4 % (36.8–100.0) and 96.9 % (15.4–100.0), respectively. Although previous studies reported on balloon volume loss during treatment [24], [25] in our practice, the balloon was constantly visible during daily cone beam CT (CBCT). None of the balloons were degraded prior to the treatment completion and none of the patients were required to undergo replanning (Fig. 4). The observation was also reported by [22] and [26]. It is possible that volume loss previously reported may be associated with the use of iodinated contrast medium [18] which may affect the balloon degradation profile. Nonetheless, later publications [27] reported on improvements made in the balloon sealing mechanism, with no further reports on balloon premature volume loss.

Our experience demonstrates that the balloon implantation procedure is easy and safe with only 3 patients complaining about grade 1 transient adverse events post-operatively. These results are comparable with reports of 94–98.8 % rates for successful spacer deployment with the hydrogel spacer [10], [16], [28], [29]. We believe that the small transperineal incision and the bevel-tipped dilator are important technical components of the balloon spacer delivery which may potentially contribute to a lesser likelihood of an inadvertent rectal perforation. Similarly, Latorzeff et al [22] reported on 86 % of the implantation procedures as easy or very easy (19 of the 22). In their report, difficulties were noted in three cases for incomplete inflation of the balloon due to resistance: difficulty crossing the perineal region and slight displacement of the balloon at the end of the inflation. All 22 patients have completed the radiation treatment usefully with a significant dosimetric gain associated with balloon (p < 0.001) with no loss of balloon volume during treatment course. The lower rates of acute GU and GI side effects that occurred within 3 months form balloon implantation are also comparable with previous reports [16], [26] and should be further evaluated with possible correlation to the concrete rectal dose sparing following the use of the balloon, and potential improvement in patient’s quality of life, as demonstrated by Hamstra et al [29] with the use of the SpaceOAR.

In our cohort there was a significant gain for the bladder at the highest radiation dose, however, the lack of effect at lower doses most likely reflects significantly lower bladder volumes in the patients following balloon implantation. Our hypothesis is that when the spacer is implanted, the isodose lines are pushed towards the rectum by decreasing the bladder dose. The use of a spacer may also improve CTV homogeneity and should reduce the maximal dose received closest to the bladder neck. This can also decrease the chance of interfraction bladder constraint violation in patients with spacers, however further prospective evaluation is needed.

Our study has several limitations. First, as a retrospective analysis there is potential for bias in the results. Second, the inclusion of patients from only one center means that our results cannot readily be generalized to other treatment centers. It is also appreciated that decisions regarding the optimal level of bladder filling are controversial. An increased amount of bladder volume in the high-dose region is usually associated with an empty bladder situation [30], [31]. The use of a rectum prostatic spacer could be a factor with potential impact in patients presenting for treatment with lower volume bladder filling [30], [31]. The relationship between bladder volume and the incidence of delayed GU sequelae are still matter of debate. Although we did not promote an active bladder volume measurement in our study, and no conclusions can be drawn from this data set, further analysis with the balloon spacer in order to obtain additional information concerning delayed OAR toxicity and data pertaining to bowel- and urinary-specific quality of life are of potential interest. The results from this open-label study are sufficiently encouraging to suggest that there would be merit in conducting further work with the balloon spacer in order to obtain additional information concerning delayed OAR toxicity and data pertaining to bowel- and urinary-specific quality of life. A prospective, randomized clinical study with the balloon spacer was conducted and completed in the EU and the US (NCT03400150) and its expected future published results may additional supportive evidence for the balloon safety and efficacy in radiation treatment for prostate cancer patients.

## Conclusions

In patients with T1-T3a PC disease who receive EBRT, balloon spacer is an effective means to substantially reduce the rectal radiation dose. Insertion of a biodegradable balloon system was technically straightforward and is a well-tolerated and effective means of securing rectal dosimetric gains at high levels of radiation dose.

Additional studies are required to establish long-term safety data and to define potential benefits in bowel, urinary, and sexual QOL parameters.

## Declaration of competing interest

The authors declare that they have no known competing financial interests or personal relationships that could have appeared to influence the work reported in this paper.
